# Summer Drink

**Published:** 1871-09

**Authors:** 


### SUMMER DRINK.

COLD black tea, with sugar and lemon-juice sufficient to make it palatable, is one of the safest, most invigorating, and cooling of all summer drinks.

The better class of Russians flavor their tea with a slice or two of lemon in each cup, instead of cream and sugar.

As a harvest drink, at the temperature of the air, the lemonaded cold tea invigorates immediately; the acid of the lemon cools the system, and promotes the action of the liver, while the sugar goes directly to the nutriment and support f the body. All cordials, beers, and ciders are exceedingly transient in their enlivening effects, and speedily and infallibly lead to the use of preparations which have a larger proportion of alcohol, and a man becomes a tippler before he is aware of it.

Tea is said to double its strength if it is ground fine like coffee, and draws in half the time if a lump of sugar, an inch or more in diameter, is put into the water.

